# Supplementary material for: Landscape of Epidermal Growth Factor Receptor Heterodimers in Brain Metastases
Source: Cancers (Basel). 2022 Jan 21;14(3):533. doi: 10.3390/cancers14030533 (PMC8833370; doi:10.3390/cancers14030533)
Supplement: Supplementary file 1 [file cancers-14-00533-s001.zip › cancers-1549716-supplementary.pdf]

cancers

MDPI

Supplementary Materials:

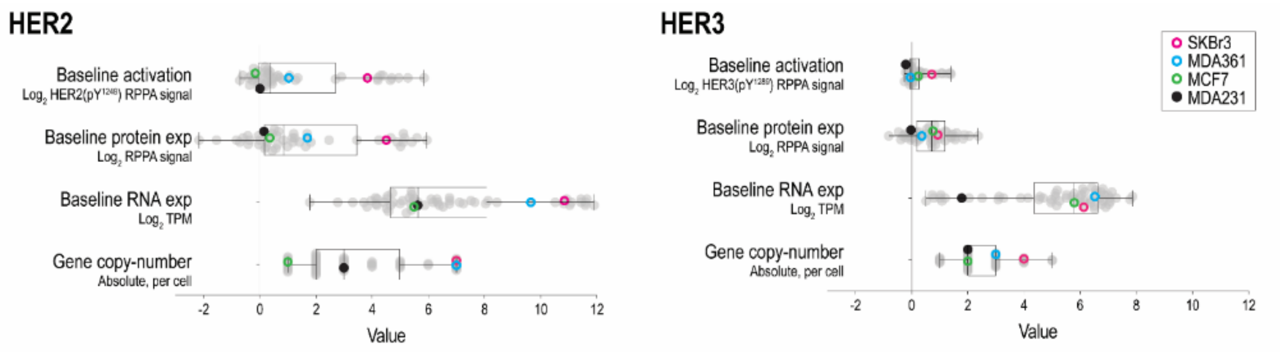

Figure S1. Cell line selection. Plots show cancer Cell Line Encyclopaedia gene copy number, RNAseq and reverse-phase protein array (RPPA) data for breast cancer cell lines, highlighting those selected for this study.

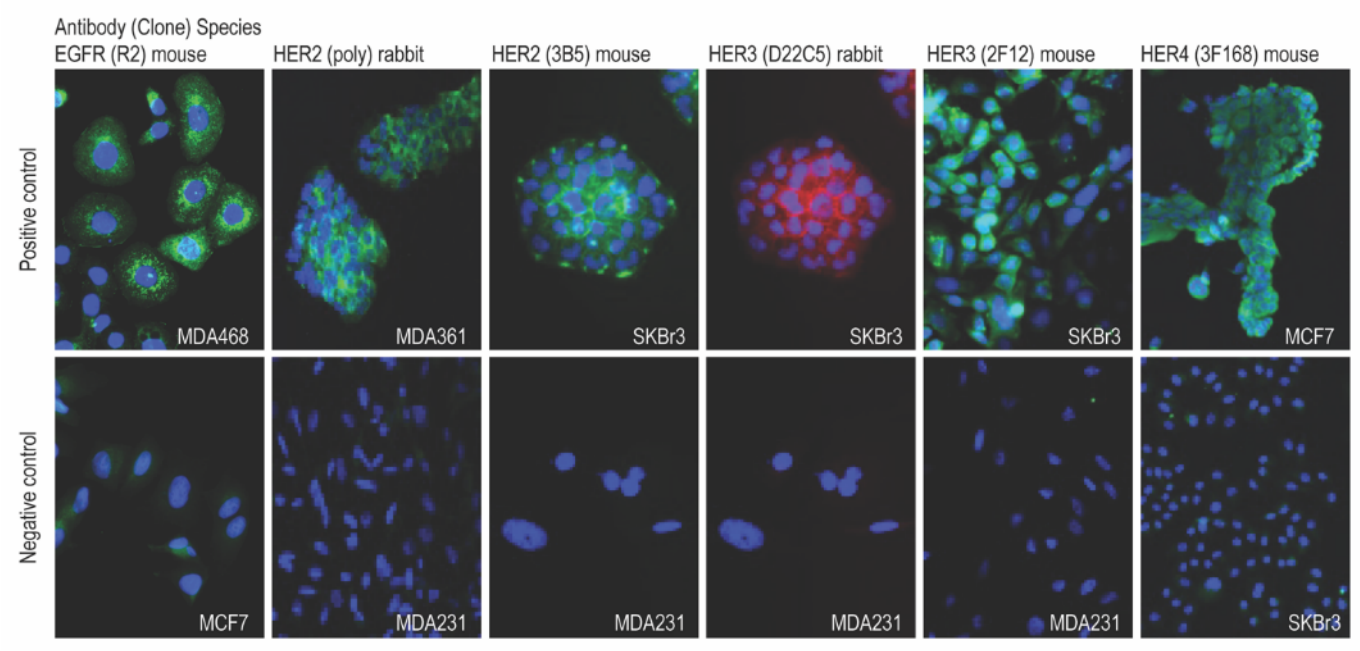

Figure S2. Validation of PLA immuno-detection conditions using immunofluorescence and fluorescent microscopy (images taken at 20x magnification). Positive and negative control cell lines were selected based on known HER family expression status.

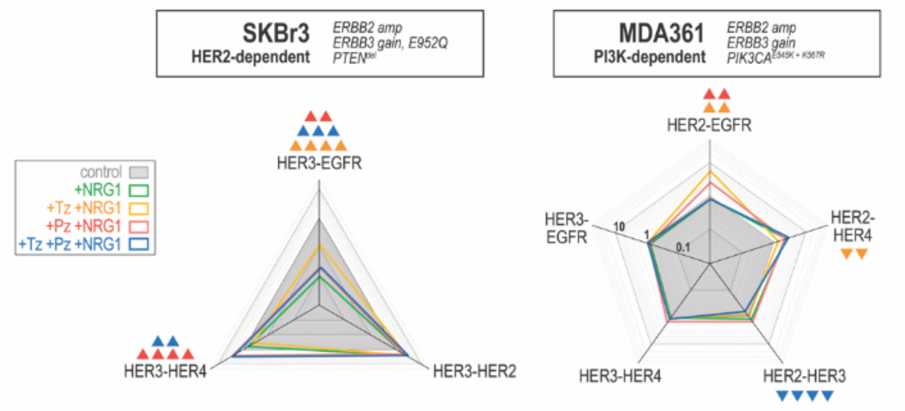

Figure S3. *In vitro* PLA data supporting Figure-4, second biological PLA replicate for the dimer complexes shown, in SKBr3 and MDA361 cells.

Supplementary Data: The following scripts can be run in Qupath 'Script Editor' as a batch.

Qupath script	Description
setImageType('FLUORESCENCE');<br>createSelectAllObject(true);<br>def path = buildFilePath(PROJECT_BASE_DIR, 'annotations')<br>def annotations = getAnnotationObjects()<br>new File(path).withObjectOutputStream {<br>it.writeObject(annotations)}	1. Annotate the image.
selectAnnotations();<br>runPlugin('qupath.imagej.detect.nuclei.WatershedCellDetection', '{"detectionImageFluorescence": 1, "requestedPixelSizeMicrons": 0.5, "backgroundRadiusMicrons": 0.0, "medianRadiusMicrons": 0.0, "sigmaMicrons": 3.0, "minAreaMicrons": 50.0, "maxAreaMicrons": 400.0, "threshold": 7000.0, "watershedPostProcess": true, "cellExpansionMicrons": 10.0, "includeNuclei": true, "smoothBoundaries": true, "makeMeasurements": true}');	2. Select the whole image annotation and perform cell detection based on nuclear stain.
name = getProjectEntry().getImageName()<br>cells = getCellObjects()<br>println name + "_" + (cells.size() + "_cells")	3. Print file name and cell count. Data is then copy into Excel sheet.
import qupath.imagej.plugins.ImageJMacroRunner<br>import qupath.lib.plugins.parameters.ParameterList<br>def params = new ImageJMacroRunner(getQuPath()).getParameterList()<br>print ParameterList.getParameterListJSON(params, ' ')<br>params.getParameters().get(' downsampleFactor ').setValue(4.0 as double)<br>print ParameterList.getParameterListJSON(params, ' ')<br>def macro = 'Stack.setChannel(3); run("Find Maxima...", "noise=1000 output=Count");'<br>def imageData = getCurrentImageData()<br>def annotations = getAnnotationObjects()<br>for (annotation in annotations) {<br>ImageJMacroRunner.runMacro(params, imageData, null, annotation, macro)}	4. Exports the annotated image to ImageJ and execute 'Find Maxima' on the designated channel which counts the PLA signals. Data is then copy into Excel sheet.
